# Biomarkers for SARS-CoV-2 infection. A narrative review

**DOI:** 10.3389/fmed.2025.1563998

**Published:** 2025-03-26

**Authors:** Sara Weronika Snopkowska Lesniak, Diego Maschio, Cesar Henriquez-Camacho, Victor Moreno Cuerda

**Affiliations:** ^1^Servicio de Medicina Interna, Hospital Universitario de Mostoles, Madrid, Spain; ^2^Facultad de Medicina, Universidad Francisco de Vitoria, Madrid, Spain; ^3^Facultad de Ciencias de la Salud, Universidad Rey Juan Carlos, Madrid, Spain

**Keywords:** biomarkers, SARS CoV-2, COVID-19, prognosis, mortality

## Abstract

COVID-19 is an infectious disease caused by SARS-CoV-2 with devastating effects on health-care systems. The magnitude of the problem has moved physicians and investigators to identify strategies to detect patients at a high risk of severe disease. The aim of this study was to identify the most relevant biomarkers in the published literature and their correlation with clinical outcomes. To this end, we performed a revision of studies that investigated laboratory abnormalities in patients with COVID-19, comparing non-severe and severe patients. Blood biomarkers were classified into five main categories: hematological, coagulation related to the liver or kidney, and inflammatory. From our analysis, the most relevant biomarkers associated with severe infection for each category were increased levels of leukocytes, neutrophils, and neutrophil-to-lymphocyte ratio; decreased platelet count; and high levels of aspartate transaminase, alanine transaminase, creatine kinase, troponin, creatinine, and blood urea nitrogen, C-reactive protein, ferritin, and IL-6. Moreover, lactate dehydrogenase and D-dimer levels were independent risk factors for death.

## 1 Introduction

In 2019, a novel coronavirus emerged in the city of Wuhan, China, and spread rapidly, causing a global pandemic responsible for almost 7,000,000 deaths globally (1).

The virus was designated severe acute respiratory syndrome coronavirus 2 (SARS-CoV-2) and the clinical manifestation of the infection coronavirus disease 2019 (COVID-19). COVID-19 presents a wide clinical spectrum, ranging from asymptomatic or mild-to-severe to lethal forms (1). Mild COVID-19 cases are characterized by a median incubation period of 5 days, followed by flu-like symptoms such as fever, myo-arthralgia, cough, and nasal congestion. Severe cases of COVID-19 are due to the development of acute respiratory distress syndrome (ARDS) or a hyper-inflammatory response known as cytokine storm, requiring intensive care unit (ICU) admission and mechanical ventilation (MV) (1, 2).

In this setting, clinical and analytical findings are crucial to identify patients at high risk, direct treatment, and improve clinical outcomes. With this goal, during the COVID-19 pandemic a large body of evidence has accumulated, both on well-known and new biomarkers.

In this review, we identified and categorized the most important biomarkers in the published literature and their correlation with clinical outcomes. Given the numerous published studies, we performed this review to synthesize the current evidence. For this purpose, we classified biomarkers according to the organ or system involved into eight categories: hematological, coagulation, cardiac, kidney, liver, inflammatory, tumor, and other biomarkers.

## 2 Search strategy and selection criteria

**Figure 1:**
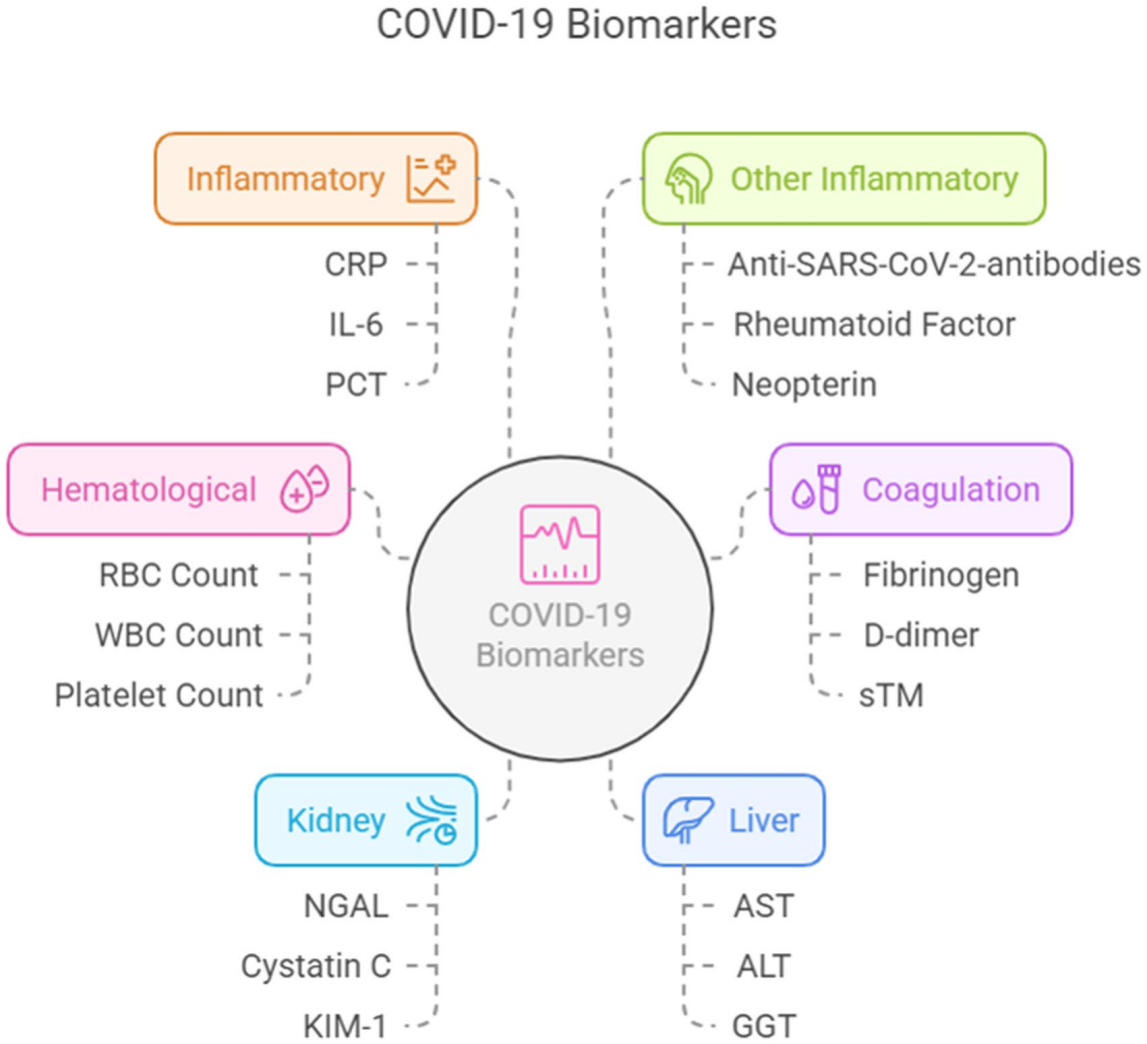
Biomarkers according to the organ or system involved. Created using Napkin AI.

Preference was given to studies regarding biomarkers in COVID-19 adult patients. There was no restriction of type of studies published within the past decade, although some older classic references were also included. We searched PubMed and WOS using the terms: “biomarkers,” “COVID-19,” “SARS CoV-2,” “prognosis,” “diagnosis,” “mortality.” We screened titles and abstracts for eligibility and potentially relevant articles were retrieved (Figure 1).

## 3 Hematological biomarkers

### 3.1 Red blood cell

The widespread distribution of red blood cells in non-survivors was the only significant disturbance in the erythroid lineage, which is consistent with previous observations of impaired synthesis of mature red blood cells due to hypoxia (1).

Furthermore, a significantly higher prevalence of elevated erythrocyte sedimentation rate (ESR, ≥30 mm/h) was observed in severe patients, especially in those who needed MV (28% vs. 15% in the overall population) (See Supplementary Table 1) (2, 3).

### 3.2 White blood cell count

White blood cell (WBC) count is a marker of systemic inflammation and immune response.

Approximately 70–80% of patients present with typical findings of viral infections, consisting of an elevation in leukocyte and neutrophil counts (leukocytosis and neutrophilia, respectively) and a concomitant decrease in lymphocyte count (lymphopenia) (4–10).

WBC and neutrophil counts were significantly higher in severe cases than in moderate cases, whereas the lymphocyte count was lower in severe patients than in moderate patients, leading to a high neutrophil-to-lymphocyte ratio (NLR). In fact, a neutrophil/lymphocyte ratio ≥ 3.5 was observed in approximately 65% of the overall COVID-19 population and in almost 80% of patients with severe outcomes (11–13).

Monocytes and macrophages are among the first cells to respond to viral infections and are responsible for the local antiviral response in the lungs. The lower peripheral blood monocyte counts observed in non-survivors of COVID-19 suggest that monocyte counts could serve as biomarkers for disease severity and outcome prediction (14).

A decrease in eosinophil count has also been recognized as a significant biomarker for COVID-19, being more prevalent in patients with a severe clinical course and may reflect an altered immune response and heightened inflammation (2, 3).

### 3.3 Platelet count

Various studies have highlighted that thrombocytopenia is more prevalent in severe COVID-19 cases than in milder ones and that the platelet count is lower in non-surviving patients than in survivors (9, 10, 15).

In patients with severe infection, thrombocytopenia was identified in up to 57.7% of patients versus 31.6% of patients with less significant COVID-19 symptoms. Moreover, a recent meta-analysis of 17 studies showed that patients with thrombocytopenia had significantly higher odds of poor outcomes (3, 9, 16, 17).

However, the results of platelet count in COVID-19 are not unanimous, and some studies have observed no significant difference in platelet count between hospitalized COVID-19 patients in the ICU and non-ICU and between survivors and non-survivors of COVID-19 complicated by ARDS (18, 19).

The platelet-to-lymphocyte ratio (PLR) is a marker of systemic inflammation and immune response. In COVID-19, an elevated PLR may reflect an imbalance between the inflammatory response (represented by platelets) and the immune response (represented by lymphocytes), and elevated PLR levels may serve as an indicator of poor prognosis in COVID-19 patients, helping to identify individuals at a higher risk of adverse outcomes (20).

### 3.4 New biomarkers

Various new biomarkers have been studied, the most relevant being MDW (Beckman Coulter), which has been reported to increase in nearly all COVID-19-infected patients, particularly in those with the worst clinical symptoms, based on non-peer-reviewed personal data recently reported in a review (21). However, MDW data should be interpreted with caution since the presence of reactive lymphocytes in COVID-19-positive patients may result in falsely elevated MDW (21).

## 4 Coagulation biomarkers

Due to intense inflammation during COVID-19 infection, some patients experience coagulation alterations leading to thrombotic complications, such as pulmonary embolism (PE) and disseminated intravascular coagulation (DIC).

Patients with severe infection show significantly higher levels of fibrinogen, D-dimer, and fibrin degradation products (FDP), which are acute-phase reactants that increase in response to inflammation. The usefulness of D-dimer can be further extended to evaluate the D-dimer to Lymphocyte ratio (DLR). The SEMI-COVID-19 Network conducted a retrospective, multicenter, observational study that included more than 10.000 patients, confirming the importance of DLR as an accessible prognostic biomarker for mortality in COVID-19 (22).

COVID-19 is known to cause endothelial dysfunction, leading to the increased expression of endothelial activation markers. Among these, the two most studied biomarkers were Soluble Thrombomodulin (sTM) and Von Willebrand Factor (VWF), which have been showed to be increased in COVID-19 patients, especially in those with severe disease. In critical patients, the ratio between high and low-molecular-weight VWF multimers was shifted toward the former, indicating the failure of metalloproteinase ADAMTS13 in converting the massive amounts of VWF released from endothelial cells (13, 19).

Platelet activation markers, such as soluble P-selectin and betathromboglobulin (BTG), have been shown to increase in critical COVID-19 cases, suggesting increased platelet activation and potential thrombotic risk, although further research is needed (19).

## 5 Cardiac biomarkers

### 5.1 Troponin

Troponin levels have been shown to be correlated with the severity of COVID-19 infection and can help identify patients at increased risk of cardiac complications and adverse outcomes, including mortality and cumulative 30-day incidence of all-cause death, as shown in many studies (23–25). In a research article published in 2020, physicians evaluated the relationship between cardiac biomarkers and thoracic computed tomography (CT) findings in COVID-19 patients, showing that COVID-19 patients with severe CT alterations had higher levels of high-sensitivity troponin I (hs-cTnI). However, the significance of troponin as a predictive marker for severity was lost when adjusting for age, sex, and other biochemical markers (26).

In summary, monitoring cTnT and cTnI levels in COVID-19 patients is crucial for assessing cardiac involvement, identifying myocardial damage, and guiding treatment decisions (27).

### 5.2 Myoglobin

Myoglobin is a protein found in skeletal and myocardial muscle, acting as an intracellular oxygen reservoir.

Elevated myoglobin was identified as an independent predictor of death in COVID-19 patients, with significantly higher levels in deceased patients than in survivors (25).

### 5.3 N-terminal pro-B-type natriuretic peptide

Many studies have demonstrated that elevated N-terminal pro-B-type natriuretic peptide (NT-proBNP) levels in patients with COVID-19 are linked to severe disease and poor outcomes. NT-proBNP levels have been shown to be a strong independent predictor of mortality and need for mechanical ventilation in COVID-19 patients. Combined with other biomarkers such as troponin, it can significantly improve risk stratification and predictive accuracy (23, 24, 26–29). In a robust retrospective Chinese study involving almost 3,000 adult Chinese hospitalized COVID-19 patients, the NT-proBNP ratio was found to be the strongest predictor of mortality using a 125 pg./mL threshold (30).

### 5.4 Creatine kinase-myocardial band

Creatine kinase-myocardial band elevation is associated with mortality, event risk, and risk stratification, although it is not as specific as other biomarkers such as hsTnI and NT-proBNP; hence, its interpretation should be performed in conjunction with other cardiac biomarkers for a more holistic evaluation of the patient’s condition (19, 24, 26, 27).

### 5.5 New biomarkers

Various new biomarkers have been proposed as indicators of myocardial damage in COVID-19, although further research is needed to fully understand their potential role in assessing cardiovascular damage. In the following paragraph, we describe the main features of the most relevant new biomarkers.

• GDF15 (Growth differentiation factor-15) is produced by cardiomyocytes and is known to have cardioprotective effects, inhibiting apoptosis in cardiomyocytes, and to be involved in cardiomyocyte hypertrophy. GDF15 has been shown to be significantly higher in patients with fatal outcomes than in those who survived (31), and its levels correlated with the length of hospitalization and were associated with ICU transfer or death (32).

- Galectin-3 is involved in myofibroblast proliferation, fibrogenesis promotion, tissue repair, and myocardial remodeling. In COVID-19 patients, elevated levels of Ga-lectin-3 have been associated with cardiac fibrosis and adverse cardiovascular out-comes (28).
- ST2 and sST2 (soluble ST2) are members of the interleukin-1 receptor family and exist in both membrane-bound (ST2L) and soluble (sST2) forms. sST2 is released in response to cardiac stress and inflammation and is considered a marker of cardiac fibrosis and remodeling (28).
- Matrix Gla Protein (MGP) is a vitamin K-dependent protein that plays a role in inhibiting vascular calcification. Decreased MGP levels due to vitamin K deficiency have been associated with increased cardiovascular calcification and cardiovascular disease risk (28).

## 6 Kidney biomarkers

Most studies have found a significant association between serum creatinine levels and COVID-19 severity (2, 18, 33–37), although a revision by Anne et al. found no significant correlation between serum creatinine levels and COVID-19 severity during hospitalization (1).

It has been demonstrated that BUN is an independent risk factor for in-hospital death during COVID-19 infection (2, 5, 36, 37).

Also, new biomarkers of tubular injury have been associated with kidney damage in COVID-19 patients, such as neutrophil gelatinase-associated lipocalin (NGAL), cystatin C, kidney injury molecule-1 (KIM-1), urine 11-dehydro-thromboxane B2, 8-hydroxy-2′-deoxyguanosine, liver-type fatty acid binding protein (L-FABP), N-acetyl-b-D-glucosaminidase, b2-microglobulin and a1-microglobulin (5).

Finally, the most common electrolyte disorders in COVID-19 are hyponatremia, hypokalemia, and hypocalcemia due to gastrointestinal and renal losses, hypoxic central damage, and the action of proinflammatory cytokines (34, 36, 38).

## 7 Liver biomarkers

Transaminases [aspartate transaminase (AST) and alanine transaminase (ALT)] and gamma-glutamyl transferase (GGT) are often elevated in patients with severe COVID-19, while alkaline phosphatase (ALP) levels are usually normal, mimicking drug-induced liver injury (2, 7, 33–35, 37, 39–41).

A study published by Gallo Marin et al. showed that patients with elevated transaminase levels have a higher probability of requiring intensive care unit (ICU) admission and mechanical ventilation (6). Both transaminases and the FIB-4 index are considered independent risk factors for severe COVID-19 (4).

Bilirubin has been shown to be a significant indicator of adverse outcomes (18, 41), although a study published by Azad Allarakia et al. found no significant association between bilirubin levels in ICU and hospitalized ward patients (14, 36).

Hypoalbuminemia is also an independent predictive factor for mortality (6, 33, 41), being higher in severe cases than in mild ones.

Another widely studied biomarker in COVID-19 is lactate dehydrogenase (LDH), an intracellular enzyme that converts lactate to pyruvate, found in most tissues of the body, but especially in the liver. It has been shown that LDH levels were significantly higher in non-survivors and patients with poor outcome (2, 15, 34, 39). There is controversy about the mechanism of LDH elevation: some physicians consider it an acute phase reactant (1, 8), while others suggest a relationship with respiratory function, being considered a predictor of respiratory failure and need for ICU admission (4, 36). Some studies have associated LDH levels with other biomarkers, creating new formulas such as the LDH/CD4 prediction model (6).

## 8 Inflammatory biomarkers

Inflammation is the key element in COVID-19 infection, and many inflammatory biomarkers have been studied, both classic and new biomarkers of inflammation (See Supplementary Table 2).

### 8.1 Classic inflammatory biomarkers

Numerous studies have investigated the relationship between COVID-19 and “classic” inflammatory biomarkers, such as C-reactive protein (CRP), procalcitonin (PCT), ferritin and various cytokines. Among these, interleukin 6 (IL-6) has been the most studied because of its role in cytokine storm, which represents the main cause of death in severe COVID-19 patients. IL-6 is a cytokine with multiple roles and can produce both pro-inflammatory and anti-inflammatory effects. Its activity is mediated by the binding to its receptor, which exists in two forms: the cell membrane IL-6 receptor (IL-6R), called “classic signaling” which mediates the anti-inflammatory effect, and the soluble form of IL-6R (sIL-6R), known as “trans-signaling,” responsible for the pro-inflammatory responses of IL-6 (13).

As shown in the PROSPERO study (1) and many other studies, CRP (4, 7, 8, 15, 34, 35, 37, 42–44), PCT (4, 15, 18, 37, 43, 45), ferritin (2, 7, 35, 43, 44), and IL-6 (8, 15, 34, 35, 42, 44, 46, 47) were all significantly correlated with the clinical outcome, being higher in severe COVID-19 infection compared to mild infection, with CRP and IL6 resulting in the best parameters to discriminate between severe and non-severe forms (48). Interestingly, a study by Li et al. (47) demonstrated that interleukin 8 (IL-8) levels correlated better than IL-6 levels with the overall clinical disease scores at different stages of the same COVID-19 patient, suggesting that IL-6 and IL-8 can be, respectively, used as biomarkers for severe COVID-19 and for COVID-19 disease prognosis (4).

Furthermore, CRP and IL-6 levels were significantly correlated with CT findings, being more elevated in patients with pulmonary infiltrates above the median (42, 46, 49).

Interferons (IFN), both alpha and gamma, have been studied in COVID-19 infection, but no significant difference in plasma levels has been shown when comparing mild and severe patients, nor when comparing mild and critical patients (42, 50).

Huang et al. (51) compared cytokine alterations in COVID-19 patients according to their evolution. Compared with patients with a persistently mild COVID-19 infection, those with a mild infection who progressed to severe had significantly increased levels of seven cytokines during the mild stage, such as hepatocyte growth factor (HGF), leukemia inhibitory factor (LIF), β-nerve growth factor (β-NGF), interleukin 3 (IL-3), interleukin 9 (IL-9), interleukin 12p40 (IL-12p40), and interleukin 17 (IL-17). In contrast, only one cytokine, TNF-α, was differentially expressed between patients with persistently severe infection and those with mild infection.

When comparing inflammatory biomarkers in COVID-19 infection and bacterial pneumonia, serum levels of CRP, C3 and C4, IL-8, IL-10, IL-12, TNF-α, and IFN-γ were significantly elevated in both groups compared to healthy controls. Furthermore, serum level of CRP and C3 were significantly higher among patients with bacterial pneumonia with respect to COVID-19 infection, while IFN-γ was higher among patients with COVID-19 than patients with bacterial pneumonia (52). Bacterial pneumonia concomitant with COVID-19 is uncommon, with a prevalence of 8% for coinfections and 20% for superinfections (53). If empiric antibiotic therapy is initiated, it is important to obtain a microbial diagnosis (e.g., culture of sputum or urinary antigens) (54).

### 8.2 Other inflammatory biomarkers

Anti-SARS-CoV-2-antibodies have been shown to be strongly associated with reduced risk of SARS-CoV-2-infection or re-infection and better outcomes, including de-creased severity of COVID-19 symptoms and lower mortality rates (See Supplementary Table 3) (55).

Among the other antibodies examined, it has been shown that the rheumatoid factor (RF) is significantly higher in severe COVID-19 than in moderate and asymptomatic cases (56).

Interleukin 17 (IL-17) has been shown to be significantly associated with a poor outcome in COVID-19 patients, including the need for mechanical ventilation and/or death within a month (57).

Presepsin (PSP) is a subtype of soluble CD14 (sCD14), which is a subtype of the Toll-like receptor (TLR) superfamily. Studies have shown that presepsin levels were significantly higher in severe and critical patients than in moderate and mild ones, in non-survivors than in survivors, and in patients requiring admission to the ICU than in non-ICU patients, and it showed a significant predictive value for 28-day mortality in COVID-19 patients, with a 775 pg./mL cutoff (58, 59).

Neopterin is a well-established immune activation biomarker whose concentration is elevated in the early phase of disorders associated with the inflammatory response. The acute phase of infection was characterized by elevated levels. Patients with poor outcomes had unchanged or decreased levels (60).

Galectine-9 belongs to the superfamily of lectin proteins that are involved in immune regulation and viral immunopathogenesis. Galectine-9 levels in plasma have been demonstrated to be significantly higher in severe COVID-19 cases than in moderate cases (61).

Adrenomedullin (ADM) is a peptide hormone with vasodilating effects that has been linked to endothelial dysfunction and the risk of organ failure in patients with sepsis. It has been observed that COVID-19 patients with poor outcome had higher levels of MR-proADM, with a cut-off level of 1 nmol/L (62, 63).

Krebs von den Lungen 6 (KL-6) is a mucin-like glycoprotein mainly expressed on the surface of type II alveolar epithelial cells and respiratory bronchiolar cells, which has been shown to be significantly higher in COVID-19 patients compared to healthy controls (64). Furthermore, serum KL-6 was linearly correlated with computed tomography lung lesion areas. Finally, no significant difference in KL-6 levels emerged between patients with severe COVID-19 and IPF, indicating that KL-6 may be used as a biomarker of pulmonary fibrosis (65).

Other novel biomarkers of inflammation and hypercoagulability are suPAR, the soluble form of urokinase-type plasminogen activator receptor (uPAR), and PIVKA-II, prothrombin induced by vitamin K deficiency or antagonist-II. The levels of PIVKA-II have been shown to be significantly higher in patients with COVID-19 than in healthy controls, whereas no significant difference was observed for suPAR (32).

MicroRNAs (miRNAs) are short and highly preserved non-coding RNA molecules of approximately 18–25 nucleotides with a role in gene expression. MiR-155 has been shown to be significantly higher in COVID-19 patients than in controls, in severe compared to moderate COVID-19 patients, and in non-survivors compared to surviving COVID-19 patients. Furthermore, miR-155 levels correlate with CT findings and are significantly higher in patients with CT ground-glass opacity and consolidation than in patients with normal chest CT scans (66). Similarly, miRNA-21-5p expression was significantly higher in COVID-19 patients than in healthy controls (37, 67).

New biomarkers are being evaluated, although high specificity is needed in some serious life-threatening infectious diseases (68) as well as COVID-19.

## 9 Variants of SARS-CoV2 and biomarkers

Different variants of SARS-CoV2 have been associated with morbidity and intensive care unit admission. More patients infected with the wild-type/alpha Variant of Concern (VOC) required intensive care than those infected with the Omicron VOC. This suggests that the wild type/alpha variant may lead to more severe diseases requiring critical care. Instead, the Omicron cohort had significantly higher levels of cardiac biomarkers (hs-TnT and NT-proBNP), indicating greater cardiac involvement, which is correlated with morbidity (69). Elevated levels of these biomarkers suggest a higher risk of cardiovascular complications associated with Omicron infections. Although the mortality rates were similar, the increased cardiac biomarkers were correlated with a higher risk of mortality, indicating that while the variants may differ in terms of severity and outcomes, they can result in serious health issues (70, 71).

## 10 Discussion and limitations

We performed a comprehensive review of studies investigating biomarkers in adult COVID-19 patients, including data from both new and classic references. Biomarkers were classified into categories, which helped in understanding how they correlated with clinical outcomes. This classification also allows for an easier comparison with previous studies that may have focused on specific categories. We identified the relevance of specific biomarkers, such as D-dimer, IL-6, and lactate dehydrogenase, and compared their predictive capabilities with findings from existing studies. One important study, the SEMI-COVID-19 network research, involved 10,000 patients supporting the significance of biomarkers as prognostic indicators for mortality and validating their findings against a broader evidence base. There is variability in the biomarker measurements across different studies. Variations in laboratory techniques, cutoff values for abnormalities, and demography may contribute to variability and dispar results and comparisons.

Another limitation is the diversity of the population. Our findings encompassed a wide range of patients, including variations in disease severity and comorbidities. This heterogeneity can influence biomarker levels and their predictive value for different subgroups of patients, limiting the generalizability and affecting external validity. Many of the studies included in this review were retrospective in nature, which can introduce biases and limit the reliability of conclusions drawn regarding causality and the temporal relationship between biomarker levels and clinical outcomes. Some confounding variables, such as the impact of overwhelming infections, treatment, and comorbidities, may influence the prognosis. These factors can skew the interpretation of biomarkers as indicators of severity or mortality risk.

Finally, new biomarkers are based on emerging data, and may require further validation. New biomarkers may not yet be fully understood and interpretations should be approached with caution. Further research is needed to establish more standardized practices for biomarker usage in COVID-19 patients.

## 11 Conclusion

- COVID-19 severity can range from mild to critical. Clinical and analytical findings are crucial to identify patients at high risk of direct treatment and improve clinical outcomes.
- The most relevant changes in hematological markers were leukocytosis and neutrophilia with concomitant lymphopenia, according to the typical features of viral infections.
- The D-dimer level was the most relevant coagulation biomarker related to COVID-19 severity.
- Elevation of organ-specific damage biomarkers is associated with poor prognosis.
- Classic inflammatory biomarkers, such as C-reactive protein, procalcitonin, IL-6 and ferritin, have been proven pivotal in assessing patient severity. New biomarkers are being studied, although more studies are needed to confirm their roles.
