# Supplementary material for: Biomarkers for SARS-CoV-2 infection. A narrative review
Source: Front Med (Lausanne). 2025 Mar 26;12:1563998. doi: 10.3389/fmed.2025.1563998 (PMC11978625; doi:10.3389/fmed.2025.1563998)
Supplement: Supplementary file 1 [file Table_1.docx]

Supplementary Material

**Table 1. Biomarkers and clinical significance**.

| **Biomarkers** | **Analytic disturbance** | **Clinical significance** | **Prognosis value** |
| --- | --- | --- | --- |
| **Hematologic biomarkers**  ESR  White blood cell count  Neutrophil count | Increased | Proinflammatory response | Yes |
|  | Increased | Proinflammatory response | Yes |
|  | Increased | Proinflammatory response | Yes |
| Lymphocyte count  Platelet count | Decreased | Proinflammatory response | Yes |
|  | Decreased | Proinflammatory response and thrombotic risk | Yes |
| Eosinophil count  **Coagulation biomarkers**  D-Dimer  Prothrombin time | Decreased | Proinflammatory response | Yes |
|  |  |  |  |
|  | Increased | Thrombotic complications and cardiac damage | Yes |
|  | Increased | Coagulopathy and cardiac damage | Yes |
| **Cardiac biomarkers**  Troponin |  |  |  |
|  | Increased | Cardiac damage/failure | Yes |
| Myoglobin | Increased | Cardiac damage/failure | Yes |
| NT-proBNP | Increased | Cardiac damage/failure | Yes |
| CK-MB | Increased | Cardiac damage/failure | Yes |
| **Renal biomarkers** |  |  |  |
| Creatinine | Increased | Renal damage/failure | Yes |
| BUN | Increased | Renal damage/failure | Yes |
| **Hepatic biomarkers** |  |  |  |
| AST | Increased | Hepatic damage/failure | Yes |
| ALT | Increased | Hepatic damage/failure | Yes |
| GGT | Increased | Hepatic damage/failure | Yes |
| LDH | Increased | Hepatic damage/failure | Yes |
| Total bilirubin | Increased | Hepatic damage/failure | Yes |
| **Inflammatory biomarkers** |  |  |  |
| CRP | Increased | Proinflammatory response | Yes |
| Procalcitonin | Increased | Proinflammatory response | Yes |
| IL-6 | Increased | Proinflammatory response | Yes |
| Ferritin | Increased | Proinflammatory response | Yes |

**Table 2. Inflammatory biomarkers in COVID-19**.

| **Biomarker** | **Correlation** | **Clinical use** |
| --- | --- | --- |
| **C-reactive protein (CRP)** | Elevated levels correlate with severe COVID-19 outcomes. | Used for risk stratification in patients. |
|  |  |  |
| **Ferritin** | High ferritin levels are associated with hyperinflammation and poor prognosis. | Elevated in severe cases; not specific to COVID-19. |
|  |  |  |
| **Interleukin-6 (IL-6)** | Critical in cytokine storm, correlates with severe disease. | Elevated levels may indicate the need for anti-inflammatory treatment. |
|  |  |  |
| **Procalcitonin (PCT)** | Differentiates bacterial infections from viral infections; elevated in co-infection cases. | Interpretation varies based on clinical context. |
|  |  |  |
| **Tumor necrosis factor-alpha (TNF-α)** | Contributes to inflammatory response; elevated levels noted in severe cases. | Needs further study to establish clinical utility. |
|  |  |  |
| **Neutrophil gelatinase-associated lipocalin (NGAL)** | Marker of acute kidney injury, linked to inflammation and severity. | Can indicate renal impairment in severe COVID-19. |

**Table 3. New biomarkers in COVID-19**.

| **Biomarker** | **Associated Findings** | **Clinical Implications** |
| --- | --- | --- |
| **Anti-SARS-CoV-2 Antibodies** | Reduced risk of infection/re-infection; lower severity and mortality rates | Indicators of potential immunity and treatment response |
| **Rheumatoid Factor (RF)** | Higher levels in severe cases compared to moderate and asymptomatic | May indicate severity and autoimmune involvement |
| **Interleukin-17 (IL-17)** | Elevated levels linked to poor outcomes; increased need for ventilation | Potential target for anti-inflammatory therapies |
| **Presepsin (PSP)** | Higher levels in severe/critical cases; predictive of 28-day mortality | Useful for risk stratification in critically ill patients |
| **Neopterin** | Elevated during early infection; decreased levels in poor outcomes | Marker of immune activation; potential prognostic indicator |
|  |  |  |
| **Galectin-9** | Higher levels in severe cases compared to moderate cases | Could serve as a biomarker for assessing COVID-19 severity |
|  |  |  |
| **Adrenomedullin (ADM)** | Higher MR-proADM levels in patients with poor outcomes; cut-off at 1285 nmol/L | Indicator of severity and risk of organ failure |
|  |  |  |
| **Krebs von den Lungen 6 (KL-6)** | Significantly higher levels in COVID-19 patients; correlates with CT lung lesion areas | Potential biomarker for pulmonary fibrosis |
| **PIVKA-II** | Higher levels in COVID-19 patients compared to healthy controls | May indicate coagulation status and severity |
| **suPAR** | No significant difference in levels observed in COVID-19 patients | Limited utility as a diagnostic marker based on current data |
| **MicroRNA-155 (miR-155)** | Significantly elevated in severe compared to moderate cases; correlates with CT findings | Potential prognostic marker for severity and outcomes |
| **MicroRNA-21-5p** | Higher levels in COVID-19 patients than in healthy controls | Indicates COVID |
